# Mitochondrial DNA and anti-mitochondrial antibodies in serum of autistic children

**DOI:** 10.1186/1742-2094-7-80

**Published:** 2010-11-17

**Authors:** Bodi Zhang, Asimenia Angelidou, Konstantinos-Dionysios Alysandratos, Magdalini Vasiadi, Konstantinos Francis, Shahrzad Asadi, Athanasios Theoharides, Kyriaki Sideri, Lefteris Lykouras, Dimitrios Kalogeromitros, Theoharis C Theoharides

**Affiliations:** 1 Laboratory of Molecular Immunopharmacology and Drug Discovery, Tufts University School of Medicine, Boston, MA, USA; 2 Department of Biochemistry, Tufts University School of Medicine, Boston, MA, USA; 3 Allergy Clinical Research Center, Allergy Section, Attikon General Hospital, University of Athens Medical School, Athens, Greece; 4 Second Department of Psychiatry, Attikon General Hospital, University of Athens Medical School, Athens, Greece; 5 Department of Pediatrics and Otorhinolaryngology, Institute of Social Health Insurance (IKA), Thessaloniki, Greece; 6 Department of Internal Medicine, Tufts University School of Medicine and Tufts Medical Center, Boston, MA, USA; 7 Department of Psychiatry, Tufts University School of Medicine and Tufts Medical Center, Boston, MA, USA

## Abstract

Autism spectrum disorders (ASD) are neurodevelopmental disorders characterized by difficulties in communication, cognitive and learning deficits, as well as stereotypic behaviors. For the majority of cases there are no reliable biomarkers or distinct pathogenesis. However, increasing evidence indicates ASD may be associated with some immune dysregulation, and may have a neuroimmune component. We recently showed that the peptide neurotensin (NT) is increased in autistic children. We now show that NT induces release of extracellular mitochondrial DNA (mtDNA) that could act as "autoimmune" trigger. We further show that serum from young autistic patients contains mtDNA (n = 20; cytochrome B, p = 0.0002 and 7S, p = 0.006), and anti-mitochondrial antibody Type 2 (n = 14; p = 0.001) as compared to normally developing, unrelated controls (n = 12). Extracellular blood mtDNA and other components may characterize an autistic endophenotype and may contribute to its pathogenesis by activating autoimmune responses.

## Background

Autism spectrum disorders (ASD) are neurodevelopmental disorders characterized by varying degrees of dysfunctional communication and social abilities, repetitive and stereotypic behaviors, as well as attention, cognitive, learning and sensory deficits [1]. The prevalence of ASD has increased impressively during the last two decades with the most current estimates being about 1/100 children [2]. In spite of numerous clues regarding the possible underlying pathophysiology, there is major disagreement among scholars as to the significance of such clues for either the pathogenesis or diagnosis of autism [1]. Moreover, there are no reliable biomarkers or effective treatment of the core symptoms [3,4].

A number of papers have suggested that ASD may be associated with some immune dysfunction in the patients [5], or the mother during gestation [6,7]. However, these papers do not provide support of direct relationship. Additional evidence suggests that ASD may have a neuroimmune component [8]. In particular, it was recently shown that the peptide neurotensin (NT) is significantly increased in young children with autistic disorder [9]. A number of studies reporting mitochondrial (mt) dysfunction in autism have focused on altered energy metabolism [10], and concluded that it may involve a subset of children with autism [11]. Mitochondria are the primary energy-generating organelles in eukaryotic cells, and they participate in multiple intracellular processes, including calcium buffering [12]. However, mitochondria evolved from bacteria that became symbiotic with eukaryotic cells and are typically prevented from being released extracellularly by autophagy [13].

We hypothesized that mitochondrial components, such as mtDNA may be released extracellularly early in life and induce an "autoimmune" response that may contribute to the pathogenesis of autism.

## Methods

### Patients

We investigated a homogeneous group of young Caucasian children with the same endophenotype. Subjects were diagnosed with autistic disorder using the ADI-R and ADOS-G scales, which have been validated in the Greek population [14]. There were no apparent clinical differences, such as gastrointestinal problems, as reported by the parents, or mitochondrial dysfunction, as indirectly suggested by normal plasma lactate/pyruvate ratio, that may have allowed separation of the autistic patients in subgroups.

Blood was obtained in the morning at least 2 hours after breakfast to minimize any diurnal or postprandial effects. Serum from patients and controls was aliquoted and frozen at -80°C until assayed. All samples were labeled only with a code number, as well as the age and sex of the respective subject. Patients were recruited from the Second Department of Psychiatry at Attikon General Hospital, University of Athens Medical School (Athens, Greece), an NIH-approved site for biological samples. Parents signed an appropriate consent form according to the Helsinki Principles. All children met ICD-10 criteria for autistic disorder. The exclusion criteria included: (1) any medical condition likely to be etiological for ASD (e.g. Rett syndrome, focal epilepsy, fragile X syndrome or tuberous sclerosis); (2) any neurologic disorder involving pathology above the brain stem, other than uncomplicated non-focal epilepsy; (3) contemporaneous evidence, or unequivocal retrospective evidence, of probable neonatal brain damage; (4) any genetic syndrome involving the CNS, even if the link with autism is uncertain; (5) clinically significant visual or auditory impairment, even after correction; (6) any circumstances that might possibly account for the picture of autism (e.g. severe nutritional or psychological deprivation); (7) active treatment with pharmacological or other agents; (8) mastocytosis (including urticaria pigmentosa); (9) history of upper airway diseases; (10) history of inflammatory diseases; and (11) history of allergies. The controls were normally developing, healthy children, unrelated to the autistic subjects, and were seen for routine health visits at the Pediatric Department of the Institute of Social Health Insurance, Thessaloniki, Greece. There were no identifiers except for age and sex. All autistic and control samples were collected over a period of six months by trained health providers. Serum was prepared immediately and stored in -80°C. All autism and control samples were then transported by the senior author on dry ice to Boston for analysis. Previous work has shown that samples are stable at this temperature. Moreover, DNA is known to be fairly stable and can be stored for months even at -20°C.

### Serum mtDNA and anti-mt antibody assays

Anti-mt antibody Type 2 (AMA-M2) was detected using a commercial EIA Kit (DRG International, Germany) [15]. Total DNA was extracted from serum samples using Qiagen DNA Micro extraction kit (Qiagen, CA). Mitochondrial specific DNA for Cytochrome B (mt-CytB) and 7S (mt-7S) was detected and quantified by Real time PCR using Taqman assay (Mt-7S: Hs02596861_s1; Mt-CYB: Hs02596867_s1; GAPDH: Hu, VIC, TAMRA, Applied Biosystems, CA) [16]. GAPDH DNA was used to exclude any genomic "contamination" [16]. Total DNA was isolated from supernatant fluids of cultured LAD2 cells using the same method.

#### Culture of LAD2 mast cells

LAD2 cells (NIH, Bethesda, MD, USA) were cultured in StemPro-34 SFM Medium (Invitrogen, Carlsbad, CA) supplemented with 100 ng/ml recombinant human stem cell factor (rhSCF, from Biovitrum, Sweden) and 1% U/ml penicillin/streptomycin. Cells were grown in an incubator in 5% CO_2 _and air environment at 37°C. All cells were used during their logarithmic growth period.

#### Statistical analysis

Samples were thawed by Dr. Angelidou. The actual qPCR and ELISA were conducted by Dr. Zhang. Data analysis was done separately by both Dr. Zhang and Dr. Angelidou under the supervision of Dr. Theoharides. The results are presented as scattergrams, with the horizontal lines indicating the means. The ASD group was compared to the control using unpaired, unequal, 2-tailed, Student's *t*-test, as well as the non-parametric Mann-Whitney *U *test. Any correlation between independent variables (mt-CytB DNA and mt-7S DNA, as well as mt-CytB DNA and AMA-M2 protein amount) was investigated using linear regression analysis. Significance of comparisons between healthy subjects and subjects with ASD is denoted by p < 0.05.

## Results

We tested serum samples from autistic patients for mtDNA (n = 20; 16 males and 4 females; mean age 3.0 ± 0.4 years) and AMA-M2 antibodies (n = 14; 11 males and 3 females; mean age 3.0 ± 0.4 years) compared to controls (n = 12; 11 males and 1 female; mean age 3 ± 1.2 years). The number of patients analyzed for AMA-M2 was smaller only because of the lack of availability of the EIA kit that would have allowed us to assay the rest of the patients.

**Figure 1:**
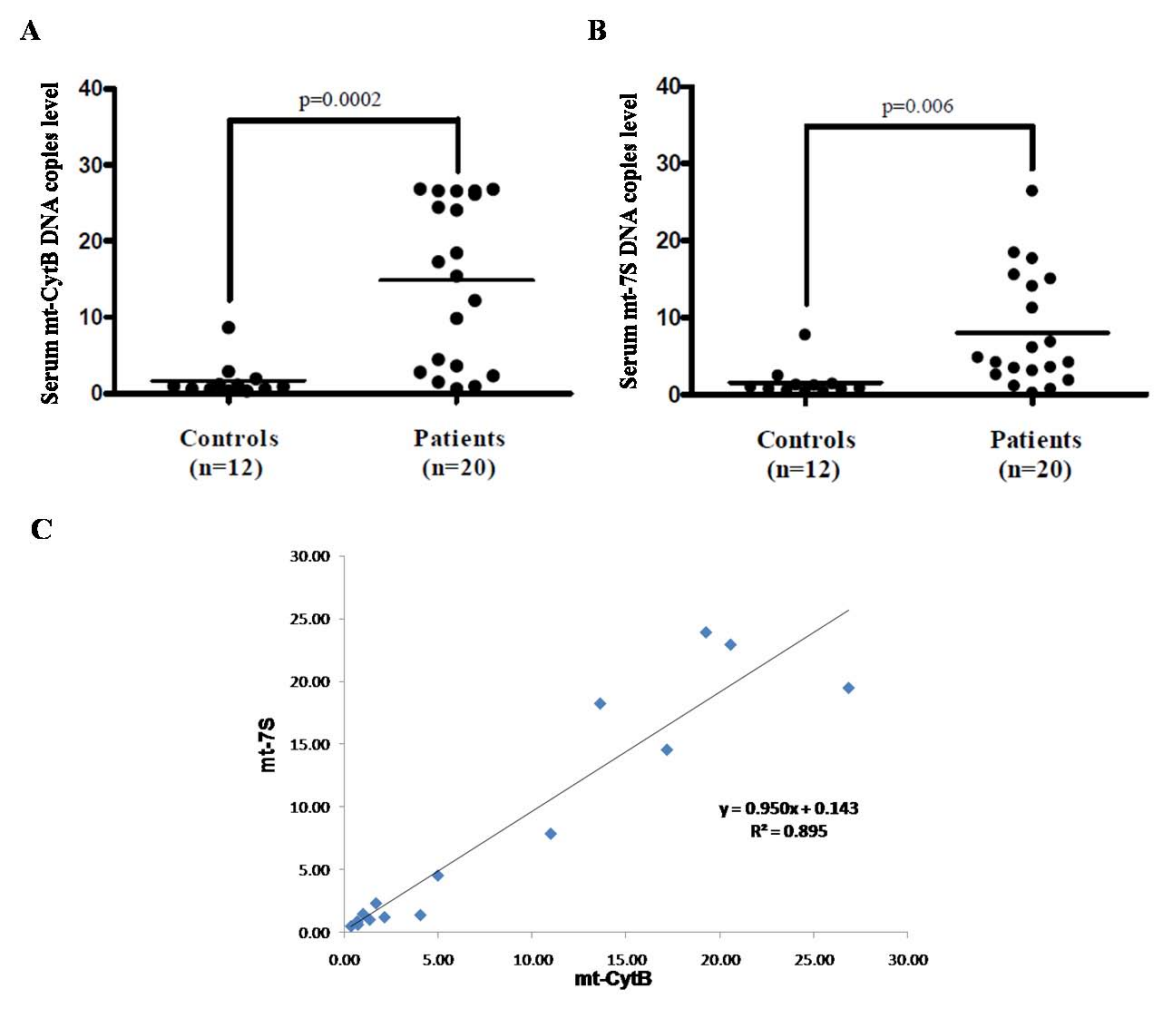
**Serum levels of (A) mt DNA Cytochrome B (CytB) and (B) mt DNA 7S in autistic patients (n = 20; 11 males and 3 females; mean age 3.0 ± 0.4 years) and controls (n = 12; 11 males and 1 female; mean age 3 ± 1.2 years)**. Genomic DNA GAPDH was undetectable, excluding the possibility of cell contamination. The horizontal lines indicate the means.(C) Linear regression analysis showing strong correlation between CytB and 7S.

**Figure 2:**
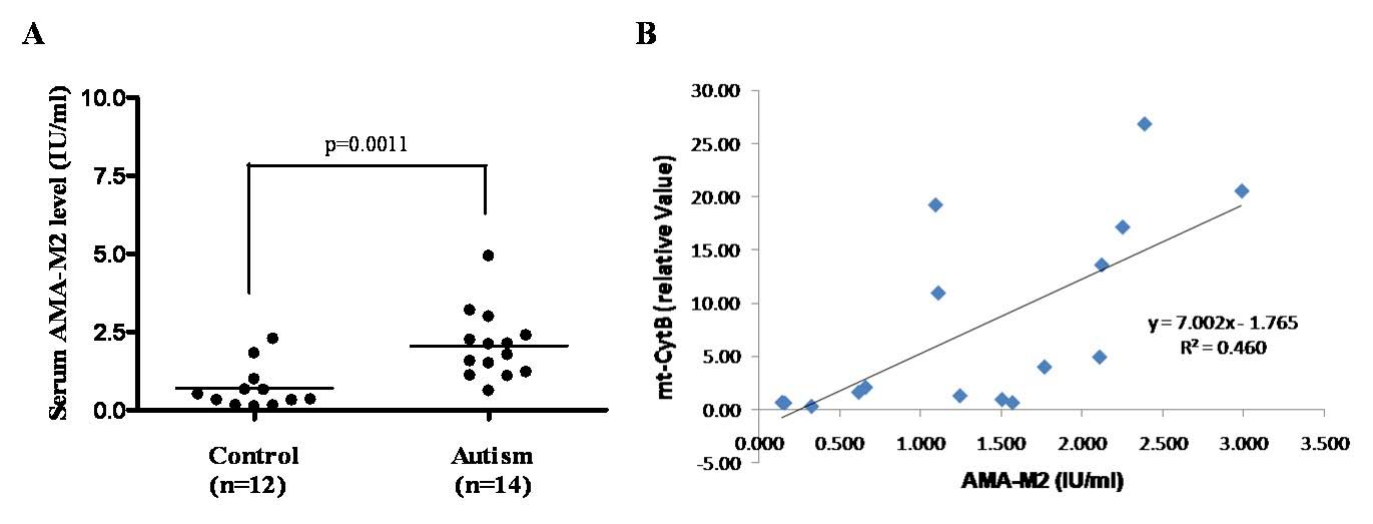
**Serum levels of anti-mt antibodies type 2 (AMA-M2) in autistic children (n = 14; 11 males and 3 females; mean age 3.0 ± 0.4 years) and controls (n = 12; 11 males and 1 female; mean age 3 ± 1.2 years)**. The horizontal lines indicate the means. AMA-M2 level were measured in International Unit (IU)/ml. (B) Linear regression analysis showing no correlation between mtDNA and AMA-M2.

We show that serum from young autistic children contains amount of mtDNA significantly higher for mt-CytB (p = 0.0002) and for mt-7S (p = 0.006) (Figure 1A and 2B). Linear regression shows an excellent correlation (R^2 = 0.89) between mt-CytB and 7S (Figure 1C. No presence of GAPDH DNA was detected indicating there was no genomic DNA release.

Serum of autistic patients also contains AMA-M2 antibodies (p = 0.001) compared to unrelated, normal controls (Figure 2). However, there was no correlation between mt-CytB DNA and AMA-M2 level (Figure 2B).

It is obvious from the scattergrams that the values corresponding to the autistic patients seem to segregate in 2 groups, indicating the possible presence of different endophenotypes. However, nothing in the clinical presentation of these children allowed for obvious separation of the subjects in subgroups.

**Figure 3:**
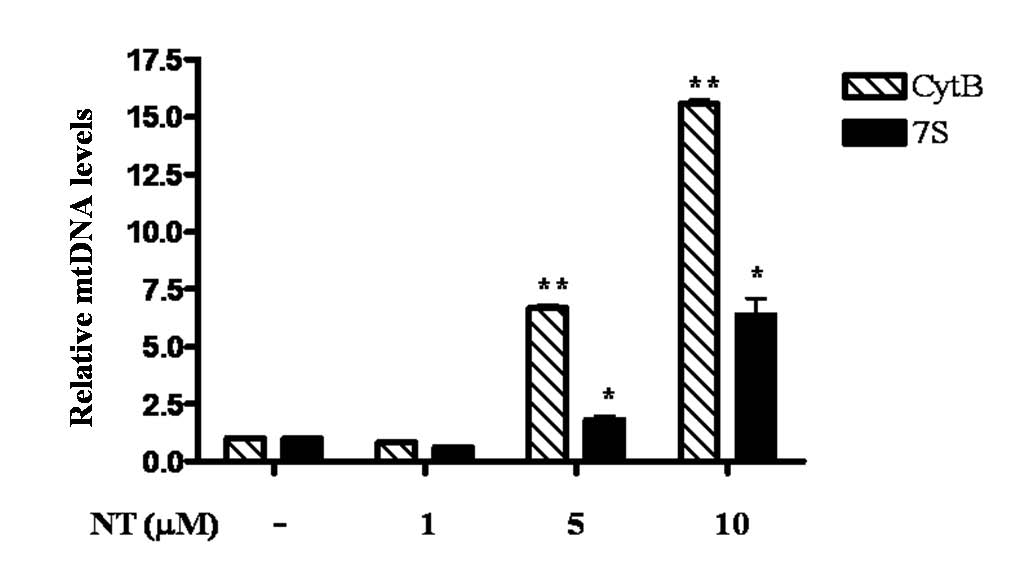
**Mitochondrial DNA detected in the supernatant fluid from NT-stimulated LAD2 cells**. LAD2 cells were stimulated with NT (1, 5, 10 μM) for 1 hr. Mitochondrial specific DNA mt-7S and mt-CytB were detected and quantified by Real time PCR using Taqman assay (Applied Biosystems, CA). Genomic DNA GAPDH was undetectable, excluding the possibility of cell contamination (*p < 0.01, **p < 0.0001).

We further hypothesized that NT may be able to trigger release of extracellular mt components from human mast cells. Stimulation of human cultured LAD2 cells with NT (1, 5 and 10 μM, for 1 h at 37°C) resulted in significant release of CytB and 7S mtDNA in the supernatant fluid (Figure 3).

## Discussion

Here we report the presence of serum extracellular mtDNA and AMA-M2 in young children with autism. Serum mtDNA has never been associated with any neuropsychiatric disease. Moreover, AMA-M2 have been clinically detected only in primary biliary cirrhosis [15]. Consequently, our findings are unique even though there are no available data as to what serum levels of either mtDNA or AMA-M2 may constitute an index of a pathological process. However, mitochondria evolved from bacteria that became symbiotic with eukaryotic cells and are typically prevented from being released extracellularly by autophagy [13]. Consequently, mtDNA released extracellularly would be extremely immunogenic. It was recently reported that damage-associated mitochondrial patterns (DAMPs), which contain mtDNA, are present in the blood of patients with trauma-induced sepsis in the absence of any apparent infection and are able to activate toll-like receptor 9(TLR9) on human peripheral polymorphonuclear leukocytes leading to release of IL-8 [16].

The presence of extacellular mtDNA in children with autism suggests that it may be one source of "autoimmune" triggers, and may potentially explain some aspects of immune dysregulation reported in autistic patients. For instance, mtDNA (or other extracellular mitochondrial components not measured in this study) could activate TLRs on immune or glial cells to release pro-inflammatory cytokines, such as IL-6, IL-8 or TNF, high gene expression of which was reported in brains of autistic children [17].

Our present results do not imply any mitochondrial dysfunction. Moreover, we cannot definitely state that subjects didn't have any mitochondrial dysfunction, since such confirmation requires extensive clinical and laboratory evaluation [18,19] that was not performed in this case. Mitochondrial dysfunction has been reported in a subset of children with autism [11,20], but apparently is not linked to altered energy metabolism [10]. Such subsets of ASD children with mitochondrial dysfunction may be more vulnerable to regression following a febrile episode [21].

The source of the extracellular mtDNA and other mitochondrial components in the serum of autistic patients is not presently known. There is no reason to suspect that these derive from apoptotic or necrotic cells because no GAPDH DNA was detected. Moreover, there is no apparent cell damage, at least outside the brain, in autism. One possibility is that mt components are secreted from immune cells, as was recently reported for activated neutrophils [22]. Another possibility could be activated tissue mast cells in the gut, where NT  [23], is abundant and may induce mucosal permeability [24]. Our current findings showing that NT can trigger mtDNA release from human mast cells is supported by our previous report that NT, found both in the brain and the gut was elevated in autistic children [9]

The present results may be limited only to autistic disorder and only to the young age of the subjects studied. Nevertheless, these results suggest that serum mt components may induce autoimmune responses, as previously reported for TLR9 activation on human peripheral polymorphonuclear leukocytes [16], and may help with early diagnosis of at least a subgroup of autistic patients.

## Competing interests

The authors declare that they have no competing interests. TCT is the inventor of patent application US 12/534,571 and provisional patent application 61/405,414 covering the diagnosis and treatment of ASD.

## Authors' contributions

BZ performed the experiments and analyzed the results. AA and KDA performed the in vitro stimulation experiment, analyzed the results and helped write the paper. MV and SA helped prepare the samples and performed computer searches. KF and KS collected all the autistic samples and reviewed the results. AT provided all the normal controls. LL and DK supervised the collection of the human samples. TCT designed the study, organized the collection of human samples, transported the samples and supervised the analysis of the results and wrote the paper. All authors have read and approved the final version of the manuscript.
